# TRIM8 downregulation in glioma affects cell proliferation and it is associated with patients survival

**DOI:** 10.1186/s12885-015-1449-9

**Published:** 2015-06-16

**Authors:** Lucia Micale, Carmela Fusco, Andrea Fontana, Raffaela Barbano, Bartolomeo Augello, Pasquelena De Nittis, Massimiliano Copetti, Maria Teresa Pellico, Barbara Mandriani, Dario Cocciadiferro, Paola Parrella, Vito Michele Fazio, Lucia Maria Cecilia Dimitri, Vincenzo D’Angelo, Chiara Novielli, Lidia Larizza, Antonio Daga, Giuseppe Merla

**Affiliations:** 1 Medical Genetics Unit, IRCCS Casa Sollievo della Sofferenza, Poliambulatorio Giovanni Paolo II, I-71013 San Giovanni Rotondo (FG), Italy; 2 Biostatistics Unit, IRCCS Casa Sollievo della Sofferenza, Poliambulatorio Giovanni Paolo II, I-71013 San Giovanni Rotondo (FG), Italy; 3 Laboratory of Oncology, IRCCS Casa Sollievo della Sofferenza, I-71013 San Giovanni Rotondo (FG), Italy; 4 Ph.D program in Experimental and Regenerative Medicine, University of Foggia, Foggia, Italy; 5 Pathology Unit, IRCCS Casa Sollievo della Sofferenza, I-71013 San Giovanni Rotondo (FG), Italy; 6 Neurosurgery Unit, IRCCS Casa Sollievo Della Sofferenza, San Giovanni Rotondo (FG), Italy; 7 Medical Genetics, Department of Health Sciences, Università degli Studi di Milano, Milan, Italy; 8 Laboratory of Medical Cytogenetics and Molecular Genetics, Istituto Auxologico Italiano, Milan, Italy; 9 Gene Transfer Lab; IRCSS Azienda Ospedaliera Universitaria San Martino-IST Istituto Nazionale per la Ricerca sul Cancro, Genoa, Italy

**Keywords:** TRIM8, Glioblastoma, miR-17, Cell proliferation

## Abstract

**Background:**

Human gliomas are a heterogeneous group of primary malignant brain tumors whose molecular pathogenesis is not yet solved. In this regard, a major research effort has been directed at identifying novel specific glioma-associated genes. Here, we investigated the effect of *TRIM8* gene in glioma.

**Methods:**

*TRIM8* transcriptional level was profiled in our own glioma cases collection by qPCR and confirmed in the independent TCGA glioma cohort. The association between *TRIM8* expression and Overall Survival and Progression-free Survival in TCGA cohort was determined by using uni-multivariable Cox regression analysis. The effect of TRIM8 on patient glioma cell proliferation was evaluated by performing MTT and clonogenic assays. The mechanisms causing the reduction of *TRIM8* expression were explored by using qPCR and in vitro assays.

**Results:**

We showed that *TRIM8* expression correlates with unfavorable clinical outcome in glioma patients. We found that a restored *TRIM8* expression induced a significant reduction of clonogenic potential in U87MG and patient’s glioblastoma cells. Finally we provide experimental evidences showing that *miR-17* directly targets the 3′ UTR of *TRIM8* and post-transcriptionally represses the expression of *TRIM8*.

**Conclusions:**

Our study provides evidences that *TRIM8* may participate in the carcinogenesis and progression of glioma and that the transcriptional repression of *TRIM8* might have potential value for predicting poor prognosis in glioma patients.

**Electronic supplementary material:**

The online version of this article (doi:10.1186/s12885-015-1449-9) contains supplementary material, which is available to authorized users.

## Background

Human gliomas are a heterogeneous group of primary malignant brain tumors, which most commonly occur in central nervous system of both children and adults [1]. Glioblastoma multiforme (GBM), the most aggressive form of glioma, exhibits advanced features of malignancy, such as rapid tumor cell proliferation, intense apoptosis resistance, florid necrosis, and robust angiogenesis [2]. The tumor properties underlie the poor clinical outcome by conferring strong resistance to chemotherapy and radiotherapy, and by promoting a neurologically debilitating course leading to death within 12–18 months post diagnosis [3]. *TRIM8* maps to chromosome 10q24.3, a region showing frequent deletion and loss of heterozygosity in human glioma [4]. TRIM8 encodes a member of the tripartite-motif-containing (TRIM) protein super family involved in a broad range of biological processes, including carcinogenesis [5]. TRIM8 interacts with and negatively regulates PIAS3, a protein inhibitor of IL-6-dependent activation of STAT3, a signaling pathway important for cancer development and progression [6]. In agreement with previously data, we recently reported TRIM8 as a new modulator of the p53-mediated tumor suppression mechanism [7]. Under stress conditions, such as UV exposure, we showed that p53 induces the expression of TRIM8, which in turn stabilizes p53 leading to cell cycle arrest and reduction of cell proliferation through enhancement of p21 and GADD45 expression [7]. Experimental evidence has outlined *TRIM8* as one of the genes which low expression level correlates with nodal metastatic progression in primary larynx squamous cell carcinoma and whose expression inhibits tumor cell colony formation *in vitro* [8]. Finally, TRIM8 deficit has been showed to impair p53-mediated cellular responses to chemotherapeutic drugs in a model of Renal Cell Carcinoma [9]. Up regulation of *miR-17,* associated with advanced tumor progression and poor overall survival of gliomas [10], has been shown to reduce the levels of *TRIM8* in primary chronic lymphocytic leukemia cells, although a direct regulation was not yet demonstrated [11].

In this study, we showed that *TRIM8* is down regulated in glioma tissues and cell lines and its expression inversely correlates with tumor grade. We found that a restored *TRIM8* expression in patient glioma cell lines suppresses the tumor growth and induced a significant reduction of clonogenic potential. Finally we showed that *miR-17* directly targets the 3′UTR of *TRIM8* and post-transcriptionally represses the expression of *TRIM8*.

## Methods

### Patients and samples

In this study we collected 70 specimens at the time of surgery from the Neurosurgery Unit IRCCS, Casa Sollievo della Sofferenza (CSS), San Giovanni Rotondo, Italy. The Ethic Committees of the IRCCS, CSS, approved this study. Prior written and informed consent was obtained from each patient in accordance with Institution Guidelines and acceptance. Upon receipt from surgery, one half of the tissue sample from the bulk of the tumor was immediately frozen in liquid nitrogen and stored at −80 °C. The second half was processed for primary tumor cultures. After surgery, glioblastoma patients were treated according to the Stupp protocol [12]. All cases were newly diagnosed gliomas, pathologically classified according to the WHO system. Clinical-pathological patients’ characteristics are reported in Additional file 1: Table S1. The median follow up time along with interquartile range (IQR) was 17.8 (IQR: 10.7-27.7) months.

### Primary tumor cell cultures generation

Glioma samples were mechanically disassociated into single cells using sterile scalpels. Tumor short-term cells suspension was cultured in a poly-lysine-coated T25 flask at 37 °C and 5 % CO2 and expanded in DMEM/F12 medium containing 10 % foetal calf serum, 1 % penicillin/streptomycin. Human U87MG glioma cell lines were grown following standard protocols. All cell culture media were purchased from Life Technologies. We were successful in obtaining primary cell culture for 45 glioma patients. Additional RNAs and DNAs from glioma-derived cell lines were reported in [13] and provided by Dr. Daga, IST, Genova. Normal human astrocyte (NHA) cell line was gently provided by Dr. Fanelli Mirko, University of Urbin, Italy.

### Quantitative real time reverse transcription-PCR (qPCR)

Total RNA was extracted using TRIZOL reagent (Life Technologies). Integrity and purity of RNAs were measured by using Agilent 2100 Bioanalyzer (Agilent Technologies) and reverse-transcribed by Quantitect Transcription kit (Qiagen), according to the manufacturer’s instructions.

Oligos for qPCR were designed using the Primer express program [14] with default parameters, with *EEF1A1* and *TBP* as references genes. qPCR reactions and calculations were made as reported in [15, 16], mRNA from NHA cell line was used as reference sample for cell line, while commercially available RNAs (Agilent Technologies) from brain of 4 healthy individuals were used in tissues gene expression studies, respectively.

### Mutational Analysis and copy number variation analysis

Genomic DNAs were extracted from fresh and frozen peripheral blood leukocytes and from cell lines using an automated DNA extractor (EZ1, Qiagen) and quantified by Nanodrop (Thermo Scientific). Sequencing of *TRIM8* coding region was performed in 70 patients. Primers were designed using the Primer 3 Output program (http://frodo.wi.mit.edu/primer3/) to amplify the 6 coding exons of *TRIM8* (RefSeq NM_030912.2) gene including the intronic flanking sequences. The amplified products were subsequently purified and sequenced as reported in [17]. All primers used in this study are available upon request. For *TRIM8* gene copy number variation analysis, four normalization assays mapping to HSA21 and four normalization DNAs were systematically included in each run as described [16]. Gene dosage segments were classified as chromosomal “gain” or “loss” if the absolute value of the predicted dosage was more than 0.75 times the interquartile range of the difference between observed and predicted values for each region. Primer pairs were designed to amplify a fragment spanning the codon 132 that encodes for the catalytic domain of IDH1. Sequence reactions were performed as reported in [17].

### miRNA expression analysis

A qPCR for *miR-17* expression in glioma cell lines and tissues was performed using TaqMan miRNA Reverse Transcription kit according to the manufacturer’s instructions. Reactions were set up in a 384-well plate using Taqman Universal PCR Master Mix (Life Technology) and run in an ABI Prism7900HT according to the manufacturer’s instructions. All qPCR experiments were performed in triplicate and the small nucleolar RNA U6 expression level was used as an endogenous control. All qPCR reagents were purchased from Life Technologies.

### Dual-luciferase reporter assay and constructs

The entire genomic region of the *miR-17-92* cluster (1094 bp) was amplified by using gene-specific primers and cloned into pcDNA3 expression vector (pcDNA3-miR-17-92). The luciferase-UTR reporter plasmid was constructed by introducing the *TRIM8* 3′-UTR into pmiR-REPORT miRNA Expression Reporter Vector System (Life Technologies). The *TRIM8* 3′-UTR sequence was amplified by PCR from HeLa cDNA. Mutagenesis was used to delete *miR-17* binding site using the QuickChange II kit (Stratagene). The pcDNA3-Flag-TRIM8-3′UTR was generated by cloning the entire open reading frame and 3′-UTR of TRIM8 amplified from HeLa cDNA into pcDNA3-Flag vector. All constructs were verified by sequencing. The reporter construct, pSV-Renilla (pRL-SV40, Promega) and *miR-17* mimic (or *miR-17-5p* hairpin inhibitor or *miR-20a* mimic, Dharmacon) or pcDNA3-*miR-17-92* were transfected into H1299, HEK293 or MCF-7 cells using Hyperfect Transfection Reagent (Qiagen) or Lipofectamine 2000 (Life Technologies). After 48 h, the cells were lysed in passive lysis buffer and assayed for both firefly and renilla luciferase activity using the Dual-GLO® Luciferase Assay System (Promega). Firefly luciferase activity was normalized to Renilla luciferase activity for each transfected well. Values are the mean ± S.E.M. of three experimental replicates from two to four independent transfections. Significance was determined by a two-tailed unpaired *t test* for means.

### MTT cell proliferation assay

Tumor cells were transfected with pcDNA3-myc-TRIM8 or empty vector using Lipofectamine LTX (Life Technologies) according to the manufacturer’s instructions and 24, 48 or 72 h after transfection MTT (3–2, 5-diphenyl tetrazolium bromide, Sigma) was added to each well of a culture plate as described previously [7]. After incubation at 37 °C for 4 h, the reaction was stopped by solubilization with isopropanol. Absorbance at a wavelength of 570 nm was measured. Each experiment was performed in triplicate.

### Clonogenic assay

Clonogenic assay was performed as previously described [18]. In brief, 5 × 10e5 U87MG and three primary glioblastoma cells were transfected with 5 micrograms of pcDNA3 vector coding for HA-TRIM8 or empty vector by using Neon Transfection Device (Life Technologies). 48 h post electroporation, the cells were analyzed for HA expression through immunofluorescence and plated in 96-well plates with a density of 0.8 cell/well. After 3 weeks, colonies defined as greater than 50 cells were counted. Each set of experiments was performed in triplicate.

### TCGA mRNA, miRNA dataset and patients’ information

mRNA, miRNA expression data and clinical information for the Glioblastoma Multiforme and Lower Grade Gliomas (LGG) datasets were downloaded from The Cancer Genome Atlas (TCGA) data portal in January and June 2013 respectively (http://cancergenome.nih.gov/) [19]. We analysed level 3 data for a total of 945 patients (567 glioblastoma and 378 LGG) with *TRIM8* expression and tumor grade information available. Data was quartile normalized and log2 transformed. Among 378 patients with LGG, 183 had WHO grade II, 195 had WHO grade III. To perform overall survival (OS) and progression-free survival (PFS) analyses, we selected 935 patients (564 glioblastoma and 371 LGG) from the 945 patients, with both outcome available information miRNA-based subtype classification. Since grade II (n = 180, from the 935 patients) and grade III (n = 191, from the 935 patients) gliomas differ substantially in prognosis, survival analyses were performed in each subgroup separately. To evaluate association between *TRIM8* CNV and tumor grade, a total of 526 patients (from the 945) with *TRIM8* CNV information available were selected (268 glioblastoma and 258 LGG). Clinical-pathological patients’ characteristics for the TCGA GBM and LGG datasets are reported in Additional file 2: Table S2.

### Statistical methods

Continuous variables were reported as mean ± standard deviation (SD) and as median along with IQR. Categorical variables were reported as absolute and relative frequencies. Normal distribution assumption was checked by means of Q-Q plot, Shapiro-Wilks and Kolmogorov-Smirnov tests.

Due to the deviation from normality distribution assumption, all statistical analyses were performed using the log-transformed expressions for *TRIM8* in glioma cell lines and tissues, whereas the square root transformation was applied for *miR-17-5p* expression levels only.

Differences of *TRIM8* in glioma tissues expression between tumour grades (WHO grades II vs. III vs. IV) were assessed using ANOVA model.

Histograms of *TRIM8* copy numbers frequency distributions were further represented with respect to tumor grades. The linear trend of *TRIM8* copy numbers across tumor grades was tested using Mantel-Haenszel Chi-Square. Boxplots of *TRIM8* expression in tissue gliomas with respect to each tumor grade and *TRIM8* copy number available were also reported. Time-to-event analyses were performed by univariable and multivariable Cox proportional hazards regression models for PFS and OS outcomes and the tertile values of *TRIM8* expression were used to categorize patients into lowest, medium and highest expression groups. Risks were reported as hazards ratios (HR) along with their 95 % confidence interval (95 % CI). Multivariable Cox proportional hazard models included the following covariates: *TRIM8* expression (in tertiles), age, the presence of *IDH1* mutation and the presence of any treatment therapy (i.e. Chemotherapy, Radiotheraphy or both). Time to disease progression was defined as the time between diagnosis and first evidence of disease progression, whereas time to death was defined as the time between diagnosis and death occurrence. Furthermore, Kaplan-Meier curves were also reported along with p-value from log-rank test. A p-value <0.05 was considered statistically significant. All statistical analyses and graphs were performed by using SAS Release 9.3 (SAS Institute, Cary, NC, USA) and R (version 2.15.2, packages: gtools, car, survival) software, respectively.

## Results

### TRIM8 is down regulated in higher-grade gliomas

**Fig. 1:**
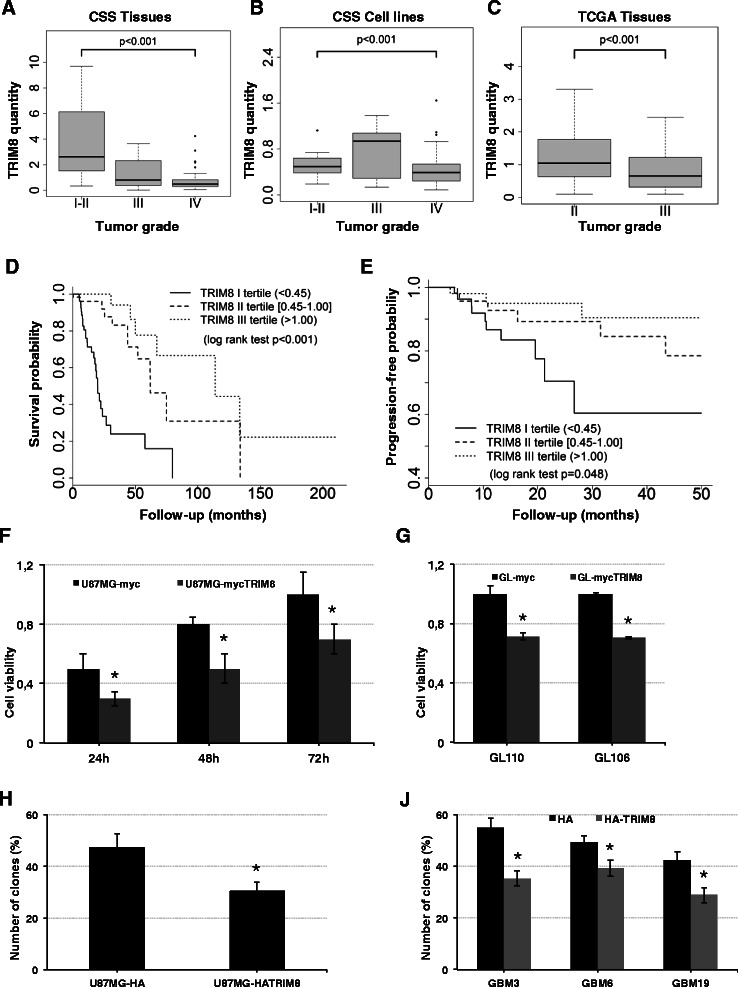
*TRIM8* is downregulated in gliomas and affects cell proliferation. (**a**-**c**) Box-plots of *TRIM8* tissues expression (**a**, **c**) and *TRIM8* cells expression (**b**) between tumor grades. qPCR was performed to measure the level of *TRIM8* transcript in a total of 70 glioma tissues and cell lines (CSS) and 378 glioma tissues specimens from TCGA LGG dataset. Kaplan-Meier curves for Overall Survival (**d**) and Progression-Free-Survival (**e**) according to TRIM8 tertiles of expression levels in patients with tumor grade III (TCGA data). (**f**-**g**) The effect of *TRIM8* expression on cell proliferation was assessed by MTT assay in U87MG glioblastoma cell lines (**f**) and in patient glioma cell lines (**g**) transfected with pcDNA3 vector expressing TRIM8 or empty vector. The Y-axis represents the absorbance value. (**h**-**j**) Effect of HA-TRIM8 expression and empty vector on *in vitro* clonogenic potential of U87MG cells (**h**) and three patient glioblastoma cell lines, GBM3, GBM6, and GBM19 (**j**). Results are reported as mean percentage of clones from three independent experiments, and error bars represent standard deviations. These experiments were repeated three independent times and similar results were obtained each time

*TRIM8* RNA expression level was determined from 71 primary glioma cell lines: 8 (WHO grade I and II), 12 (III), 51 (IV) and 70 tumor tissues: 16 (WHO grade I and II), 10 (III), 44 (IV). *TRIM8* relative expression ranged from 0.09 to 1.65 in glioma cell lines and 0.01-16.13 in tumor tissues, respectively. We found a statistically significant lower *TRIM8* relative expression in grade IV, when compared with grades II and III (p < 0.001) in both tumor tissues and cell lines (Fig. 1a and b). In tumor tissue glioma, median *TRIM8* relative expressions were 0.49 in GBM (IQR: 0.30-0.82), 0.81 in WHO grade III (IQR: 0.37-2.32) and 2.61 in WHO grade I-II (IQR:1.52-6.14) glioma (Fig. 1a). We next examined the transcriptional profile of TRIM8 in an independent cohort, i.e. the TCGA cohort, detecting a significant lower expression of TRIM8 in grade III glioma (Median:0.65, IQR:0.32-1.23) as compared with grade II glioma (Median: 1.05, IQR 0.63-1.79) (Fig. 1c). GBM expression profile data were not analyzed and compared as they were generated by a different platform compared to LGG (https://tcga-data.nci.nih.gov/tcga/).

### Low TRIM8 tissues expression level is associated with unfavorable clinical outcome in WHO grade III gliomas

We evaluated the association between *TRIM8* expression and Overall Survival and Progression-free Survival in 180 grade II, 191 grade III and 564 grade IV gliomas of TCGA cohort. Tertiles of *TRIM8* expression were calculated within each tumor grade, separately.

In WHO grade III tumors, univariable Cox regression analysis revealed a significant increase of mortality risk in patients with the lowest *TRIM8* expression levels (<0.45, first tertile) as compared to those with the highest levels (>1.00, third tertile). Indeed, we estimated a HR of 0.09 (95 % CI: 0.04-0.22, p < 0.001) and 0.18 (95 % CI: 0.08-0.36, p < 0.001) comparing the third and the second tertiles with the first one, respectively. Such results were confirmed in the multivariable Cox regression analysis comparing the third tertile to the first tertile (HR = 0.23, 95 % CI: 0.10-0.54, p = 0.001) (Fig. 1d). Moreover, univariable Cox analysis also evidenced a significant increase in the risk of disease progression in patients with the lowest *TRIM8* expression levels with respect to those with the highest level (third vs. first tertiles HR = 0.20, 95 % CI: 0.05-0.76, p = 0.018; second vs. first tertiles HR = 0.43; 95 % CI: 0.15-1.22, p = 0.114) (Fig. 1e). No significant differences were found among tertiles group in multivariable analysis, probably due to the low number of progression events, with a consequent loss of statistical power. Overall these data suggested that TRIM8 expression levels might represent an independent predictor of survival in WHO grade III gliomas. No statistically significant association with OS and PFS were found in WHO grade II gliomas and GBM.

### TRIM8 reduces cell proliferation in glioblastomas

To evaluate the effect of TRIM8 in glioma cell proliferation, we transfected U87MG glioma cells with a vector expressing TRIM8 and measured cell viability by MTT assay at 24, 48 and 72 h post transfection, respectively. Results showed that TRIM8 overexpression significantly reduced cell proliferation by about 25 % (Fig. 1f, p < 0.05, Additional file 3: Figure S1A). We next analyzed the biological effect of TRIM8 expression by restoring expression levels of *TRIM8* in two representative GBM patients cell lines. MTT assay confirmed that, in the presence of TRIM8, the rate of cell proliferation is significantly reduced by about 30 % (Fig. 1g, p < 0.01).

We therefore investigated whether overexpression of TRIM8 affects clonogenic potential of glioma cells as an indirect index of their tumorigenic potential. U87MG glioma cells, transfected with expressing TRIM8 or control vector (with transfection efficiency >80 %, measured by immunofluorescence, data not shown), were plated at limiting dilution and the formation of large colonies (>50 cells) was assessed after 3 weeks. The enforced expression of TRIM8 induced a significant reduction of clonogenic potential (p = 0.014) from the average number of 47.9 % colonies in control-transfected cultures to 30.4 % in TRIM8 transfected cells (Fig. 1h). Consistently these results were confirmed in three primary glioblastoma cell lines (GBM3, GBM6, and GBM19) with an average number of 55 %, 49.3 %, and 42.5 % colonies in control cultures, respectively, and 35.3 %. 39.3 %, and 28.9 %, respectively, in glioma TRIM8 expressing cells (Fig. 1j, Additional file 3: Figure S1B).

### Loss TRIM8 copy number in glioma

**Fig. 2:**
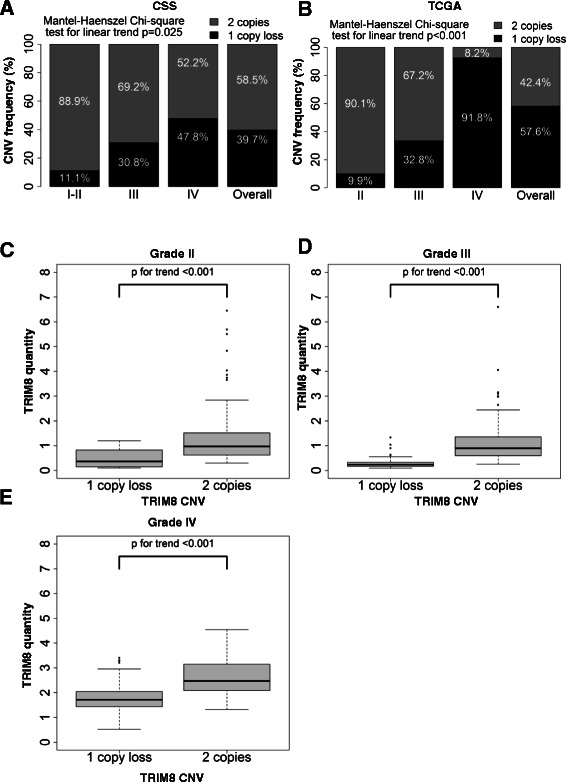
Loss of *TRIM8* copy number in gliomas. **a** Frequency distribution of *TRIM8* copy number, within each tumor grade, along with p-value from Mantel-Haenszel Chi-Square test for linear trend. *TRIM8* copy number in the gliomas was explored by using qPCR on DNA extracts from patient glioma cell lines and peripheral blood. **b** Frequency distribution of *TRIM8* CNV by tumor grade (TCGA data). **c**-**e** Boxplots of *TRIM8* expression in tissue gliomas by *TRIM8* copy number for each tumor grade separately (TCGA data)

To investigate the mechanisms that may cause the reduction of *TRIM8* expression in glioma we sequenced the coding and untranslated regions of *TRIM8* in 70 patients detecting any likely pathogenic variant (data not shown). Then we sought to determine *TRIM8* copy number by qPCR. *TRIM8* copy number results were obtained in 68 out of the70 patients from out cohort. We detected a somatic heterozygous deletion encompassing the full *TRIM8* gene in 27 out of 68 (39.7 %) analyzed glioma cells. We found a linear trend of *TRIM8* copy number across tumor grades (Fig. 2a, p = 0.025). Specifically, a heterozygous deletion of *TRIM8* was observed in approximately 11.1 % of gliomas of WHO grades I and II, 30.8 % of grade III, and 47.8 % of glioblastomas (Fig. 2a).

To confirm this evidence, we used the available *TRIM8* copy number information from the 526 of TCGA cohort, detecting a heterozygous deletion of *TRIM8* gene in 303 out of 526 (57.6 %) analyzed glioma tissues. Specifically, the loss of one copy of TRIM8 gene was observed in approximately 9.9 % of gliomas of grade II, 32.8 % of grade III, and 91.8 % of glioblastomas (Fig. 2b, p < 0.001). Loss of *TRIM8* copy number was found significantly associated to *TRIM8* low expression level in TCGA group (Fig. 2c-e).

### miR-17 regulates TRIM8 expression at the post-transcriptional level

**Fig. 3:**
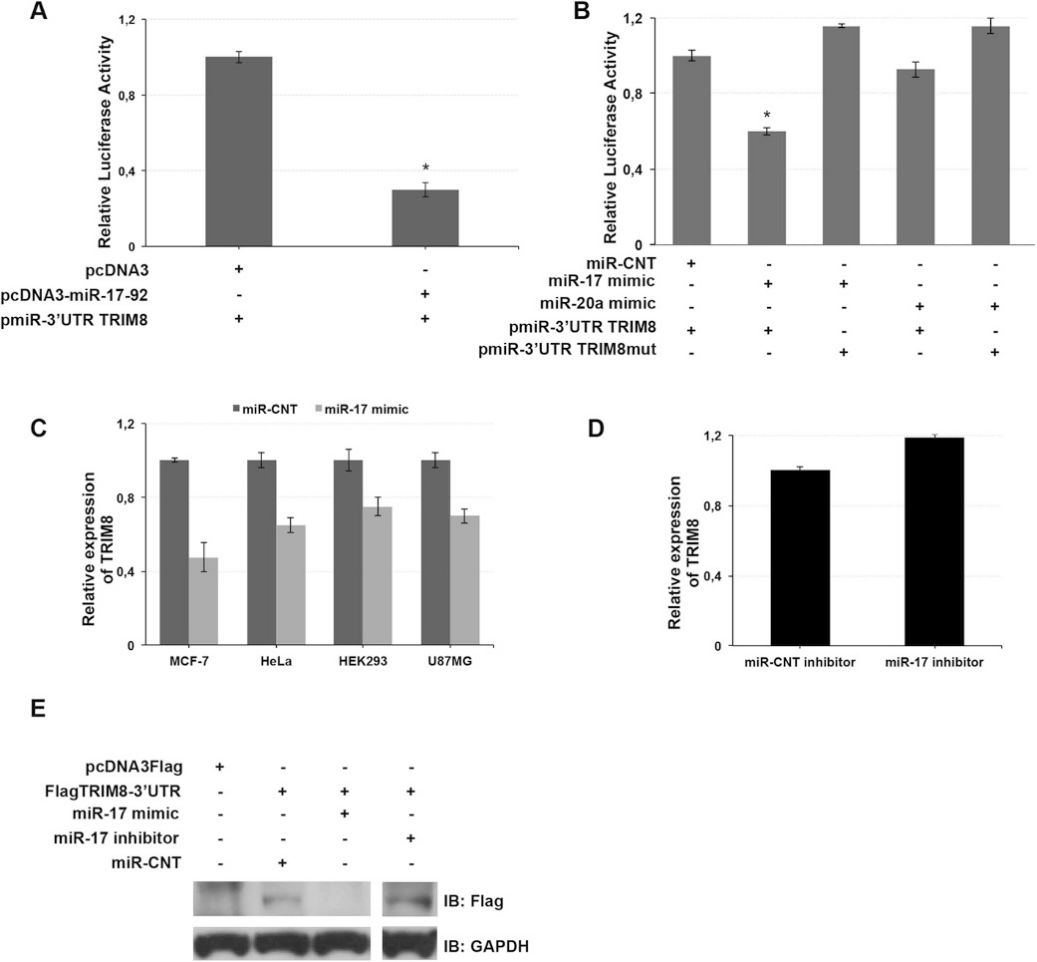
miR-17 regulates TRIM8 expression at the post-transcriptional level. **a** HEK293 cells were co-transfected with a reporter construct carrying the 3′ UTR of *TRIM8* and a pcDNA3-miR-17-92 or pcDNA3 empty vector. Luciferase activities were measured and normalized to the level of control Renilla luciferase. **b** HEK293 cells were co-transfected with reporter constructs carrying the 3′UTR of *TRIM8* or 3′ UTR of *TRIM8* containing mutated *miR-17* complementary site and a synthetic mimic of *miR-17*, mimic of *miR-20a*, or miR-control (miR-CNT). **c** Detection of *TRIM8* endogenous expression by qPCR in MCF-7, HeLa, HEK293, and U87MG cell lines transfected with *miR-17* mimic or *miR*-control. **d***TRIM8* endogenous expression in MCF-7 cell lines transfected with *miR-17* inhibitor or *miR*-control inhibitor. **e** Immunoblotting analysis by using indicated antibodies on whole protein lysate from U87MG transfected with a construct carrying the ORF and 3′UTR of TRIM8 or the ORF and 3′ UTR of TRIM8 containing a deletion of miR-17 complementary site and a miR-17 mimic, miR-17 inhibitor, or miR-control

We screened the 3′UTR of *TRIM8* by bioinformatic tools to search for putative miRNAs binding sites. As a result, we found that the 3′UTR of *TRIM8* contains two putative conserved *miR-17* and *miR-20a* binding sites. To experimentally *in vitro* test whether *TRIM8* is directly targeted by *miR-17* and *miR-20a*, the 3′UTR of *TRIM8* gene carrying wild type and mutated miRNA seed was inserted into a luciferase reporter vector and the entire genomic coding region of the *miR-17-92* cluster was cloned into pcDNA3 expression plasmid. We observed a luciferase reporter activity reduced by 60 % in HEK293 cells co-transfected with the 3′UTR *TRIM8* and miR-17-92 expressing vectors (p < 0.05, Fig. 3a).

Next, to test whether *miR-17* or *miR-20a* directly target the 3′UTR of *TRIM8*, HEK293 cells were co-transfected with 3′UTR *TRIM8* reporter construct along with a synthetic mimic of *miR-17* or of *miR-20a*. As shown in Fig. 3b the overexpression of *miR-17* significantly reduced the luciferase activity of the vector containing the 3′UTR of *TRIM8* when compared to the control (p = 0.005). Consistently, deletion of the binding site abrogated this effect (Fig. 3b). Collectively, these results indicated that the 3′UTR of *TRIM8* is targeted by miR-17.

Further we explored whether this regulation occurred also for endogenous *TRIM8*. In keeping with our luciferase data, qPCR results showed a negative correlation between *miR-17* and *TRIM8* expression levels in several human cell lines including breast and glioma cells (Fig. 3c). On the other hand, MCF-7 cells transfected with *miR-17* inhibitor had a slight increased expression of *TRIM8* mRNA when compared with *miR* control inhibitor (Fig. 3d). Finally, we found a slight negative correlation between *TRIM8* and *miR-17* expression in glioma tissue in grade II (r = −0.172, p = 0.020) and glioblastoma (r = −0.088, p = 0.038) patients from TCGA cohort, supporting the assertion that *miR-17* might contribute to modulate *TRIM8* expression in gliomas (data not shown).

In order to assess whether *miR-17* also regulates protein level of TRIM8 in glioma cells, we co-transfected U87MG cells with a pcDNA3-FLAG vector containing the ORF and 3′UTR of *TRIM8* (hereafter named TRIM8-3′UTR), along with synthesized *miR-17* or *miR-17* inhibitor, respectively. The level of TRIM8-tagged fusion protein was reduced by *miR-17* mimic and enhanced by *miR-17* inhibitor (Fig. 3e). Overall these evidences demonstrate that *miR-17* regulates *TRIM8* expression at both transcriptional and post-transcriptional level by directly binding the 3′UTR region of *TRIM8*.

## Discussion

Human gliomas are the most common and lethal neurological malignancies in adults. Our study is the first that investigates the role of the E3 ubiquitin ligase TRIM8 in glioma. We presented experimental evidences showing that i) *TRIM8* expression level is significantly decreased in glioma and is inversely associated with glioma WHO grades in both our own cases collection and in the independent TCGA glioma cohort, ii) lower *TRIM8* tissues expression level is an independent predictor of risk of death in WHO grade III tumours (TCGA dataset), and iii) the overexpression of *TRIM8* suppresses cell growth and induces a significant reduction of clonogenic potential in both U87MG glioblasto ma and patient’s primary glioma cell lines.

We investigated the molecular mechanism that can explain the observed *TRIM8* expression decrease. Our experimental data suggested that the inactivation of *TRIM8* in glioblastoma cells may occurs primarily through the loss of gene copy number. Moreover we showed that *TRIM8* expression is regulated by *miR-17* at transcriptional and post-transcriptional level. Accumulating data have indicated that the inhibition of *miR-17* significantly reduce cell viability and increases apoptotic activity in glioma cell lines and the upregulation of this miRNA is associated with advanced tumour progression and poor overall survival of gliomas [20]. Our preliminary results suggest the existence of a feedback circuit involving *miR-17* and *TRIM8* for glioma pathogenesis.

Our data corroborate mounting evidence showing a downregulation of *TRIM8* in different cancers and its role in controlling cell growth. In fact, recent data showed that *TRIM8* low expression level correlates with nodal metastatic progression in primary larynx squamous cell carcinoma and whose expression inhibits tumour cell colony formation *in vitro* [8, 21]. Yet, we previously ascertained that TRIM8 is an important component in controlling the molecular switch that directs p53 toward transcriptional activation of cell cycle arrest genes such as p21 and GADD45 [7]. Finally, downregulation of *TRIM8* was shown to impair the p53-mediated cellular responses to chemotherapeutic drugs in renal cell carcinoma [9].

In our study, the analysis of the TCGA datasets evidenced a significant increase in the risk of death and disease progression in WHO grade III tumours with the lowest *TRIM8* expression levels compared to those with the highest level. These results suggest that loss of TRIM8 expression may be necessary for the transition to a more aggressive phenotype typical of WHO grade III gliomas as compared with WHO grade II tumours, but it may have less effect on the clinical behaviour of GBMs, suggesting a likely role of *TRIM8* gene in gliomagenesis and disease progression.

Many examples are showing that TRIM family members, through their E3 ubiquitin ligases activity, are involved in oncogenic processes such as cell proliferation and apoptosis [5, 22]. For instance, TRIM32 induces tumour necrosis factor (TNF)-mediated apoptosis through its direct interaction and ubiquitylation of X-linked inhibitor of apoptosis (XIAP) [23]. The E3 ubiquitin ligases activity of TRIM13, TRIM19, TRIM24 and TRIM28 regulate the stability or transcriptional activity of p53 that leads to the control of apoptosis and DNA damage response [24]. Finally TRIM19, TRIM25, and TRIM68, by regulating the activation of nuclear and hormone receptors, play a key role in the progression of leukaemia, as well as the development of breast and prostate cancer [21]. Based on this group of experimental data, we can hypothesize that defects in TRIM8 E3 ligase activity in glioma cells might promote carcinogenesis and cancerous growth by contributing to oncogenes stabilization and/or enhancing tumour suppressors degradation.

## Conclusions

Our data give preliminary evidences that *TRIM8* may participate in the carcinogenesis and progression of glioma and that the transcriptional repression of *TRIM8* might have potential value for predicting poor prognosis in glioma patients. Moreover, our preliminary results suggest that TRIM8 and *miR-17* may be part of a same circuit involved in glioma pathogenesis, although further experiments are needed to confirm the involvement of this modulation in gliomagenesis.

## Additional files

Additional file 1: Table S1. Clinical-pathological patients’ characteristics according to WHO grade classification.
Additional file 2: Table S2. Clinical-pathological patients’ characteristics according to WHO grade classification (TCGA).
Additional file 3: Figure S3. TRIM8 expression level in transfected U87MG and GBM cells. mRNA and protein level of TRIM8 in U87MG (A) and GBM (B) cell lines transfected with a vector expressing TRIM8 or an empty vector was detected by qPCR (A-B) and Western Blot (A-B), respectively, 72 h post transfection.

## Abbreviations

GMB: Glioblastoma multiforme
TRIM: Tripartite motif-containing protein
CSS: Casa Sollievo della Sofferenza
NHA: Normal human astrocyte
qPCR: quantitative polymerase chain reaction
MTT: 3-2, 5-diphenyl tetrazolium bromide
TCGA: The Cancer Genome Atlas
LGG: Lower Grade Gliomas
PFS: Progression-free survival
OS: Overall Survival
HR: hazards ratio
CNV: Copy Number Variations
WHO: World Health Organization
ORF: Open Reading Frame
UTR: Untranslated Region
