# Supplementary material for: TRIM8 downregulation in glioma affects cell proliferation and it is associated with patients survival
Source: BMC Cancer. 2015 Jun 16;15:470. doi: 10.1186/s12885-015-1449-9 (PMC4468980; doi:10.1186/s12885-015-1449-9)
Supplement: Additional file 1: Table S1. — Clinical-pathological patients’ characteristics according to WHO grade classification. [file 12885_2015_1449_MOESM1_ESM.docx]

**Additional file 1: Table S1** – Clinical-pathological patients’ characteristics according to WHO grade classification

| **Variable** | **Category** | **All patients**  **(N=70)** | **Grade I-II**  **(N=16)** | **Grade III**  **(N=10)** | **Grade IV**  **(N=44)** |
| --- | --- | --- | --- | --- | --- |
| Age at diagnosis (years) | Mean±SD | 54.97±14.09 | 40.50±10.84 | 53.60±13.14 | 60.55±11.46 |
|  | Median (IQR) | 57.50 (43.00-67.00) | 38.00 (33-48) | 53.50 (43-67) | 61.5 (54.5-67) |
|  | Range | 23-78 | 23-60 | 33-67 | 30-78 |
| Males (n,%) |  | 46 (66.67) | 13 (86.67) | 5 (50.00) | 28 (63.64) |
| Number of interventions (n,%) | Missing values (N) | 4 | 1 | 1 | 2 |
|  | 1 | 58 (87.88) | 14 (93.33) | 9 (100.00) | 35 (83.33) |
|  | 2 | 8 (12.12) | 1 (6.67) | 0 (0.00) | 7 (16.67) |
| Therapy (n,%) | Missing values (N) | 12 | 6 | 1 | 5 |
|  | None | 5 (8.62) | 0 (0.00) | 0 (0.00) | 5 (12.82) |
|  | Radiotherapy + Chemioteraphy | 53 (91.38) | 10 (100.00) | 9 (100.00) | 34 (87.18) |
| MGMT | Mean±SD | 117.59±577.71 | 40.25±53.61 | 30.87±37.53 | 159.78±712.49 |
|  | Median (IQR) | 3.12 (0.17-60.10) | 10.48 (0.58-70.60) | 17.92 (4.55-42.71) | 0.88 (0.00-50.10) |
|  | Range | 0.00-4362.23 | 0.00-185.86 | 0.78-101.31 | 0.00-4362.23 |
|  | N° methylated patients ≥ 0.219 (n,%) | 43 (61.43) | 11 (68.75) | 6 (60.00) | 26 (59.09) |
| IDH1 (n,%) | Missing values (N) | 9 | 1 | 3 | 5 |
|  | WT | 48 (78.69) | 5 (33.33) | 5 (71.43) | 38 (97.44) |
|  | R132 | 13 (21.31) | 10 (66.67) | 2 (28.57) | 1 (2.56) |
| NANOG relative expression in glioma tissue | Mean±SD | 16.55±22.21 | 14.73±24.83 | 23.72±24.69 | 15.54±20.92 |
|  | Median (IQR) | 7.25 (2.89-20.01) | 5.33 (2.71-17.14) | 15.83 (7.22-31.35) | 8.03 (2.46-21.72) |
|  | Range | 0.29-96.32 | 1.48-96.32 | 2.17-84.01 | 0.29-89.51 |
| TRIM8 relative expression in glioma tissue | Mean±SD | 1.76±2.62 | 3.70±2.82 | 1.41±1.31 | 1.13±2.46 |
|  | Median (IQR) | 0.66 (0.35-2.18) | 2.61 (1.52-6.14) | 0.81 (0.37-2.32) | 0.49 (0.30-0.82) |
|  | Range | 0.01-16.13 | 0.35-9.70 | 0.01-3.65 | 0.06-16.13 |
| TRIM8 relative expression in glioma cell lines | Mean±SD | 0.41±0.29 | 0.48±0.18 | 0.18±0.07 | 0.41±0.32 |
|  | Median (IQR) | 0.33 (0.23-0.52) | 0.50 (0.41-0.55) | 0.18 (0.14-0.23) | 0.32 (0.23-0.52) |
|  | Range | 0.09-1.65 | 0.19-0.74 | 0.14-0.23 | 0.09-1.65 |
| TRIM8 Copy-Number-Variant (n° copies) | Missing values (N) | 44 | 10 | 8 | 26 |
|  | Loss (1 copy) | 8 (30.77) | 0 (0.00) | 1 (50.00) | 7 (38.89) |
|  | Norm (2 copies) | 18 (69.23) | 6 (100.00) | 1 (50.00) | 11 (61.11) |
| miR-17 relative expression in glioma tissue | Mean±SD | 1.38±1.86 | 1.43±2.05 | 2.19±3.55 | 1.14±0.89 |
|  | Median (IQR) | 0.86 (0.37-1.63) | 0.70 (0.31-1.08) | 0.91 (0.48-2.12) | 0.96 (0.38-1.55) |
|  | Range | 0.00-12.06 | 0.13-7.29 | 0.33-12.06 | 0.00-3.29 |
| miR-17 relative expression in glioma cell lines | Mean±SD | 1.96±1.68 | 2.33±0.43 | 2.08±0.00 | 1.86±1.92 |
|  | Median (IQR) | 1.98 (0.42-2.81) | 2.30 (1.97-2.69) | 2.08 (2.08-2.08) | 1.42 (0.15-3.09) |
|  | Range | 0.01-5.96 | 1.90-2.81 | 2.08-2.08 | 0.01-5.96 |
| Time to progression (months) | Mean±SD | 15.84±15.45 | 30.94±17.93 | 19.34±18.70 | 9.55±8.34 |
|  | Median (IQR) | 12.70 (4.77-19.10) | 31.80 (17.33-48.07) | 14.00 (6.40-17.77) | 6.07 (3.88-15.92) |
|  | Range | 0.00-64.10 | 0.83-60.50 | 3.83-64.10 | 0.00-40 |
| Overall follow-up time (months) | Mean±SD | 22.76±19.89 | 37.73±24.44 | 31.90±27.98 | 15.24±10.09 |
|  | Median (IQR) | 17.83 (10.73-27.67) | 32.18 (21.25-49.77) | 23.27 (15.13-46.03) | 13.00 (8.78-20.08) |
|  | Range | 1.43-105.93 | 1.83-105.93 | 3.83-100.57 | 1.43-51.40 |
