# Supplementary material for: TRIM8 downregulation in glioma affects cell proliferation and it is associated with patients survival
Source: BMC Cancer. 2015 Jun 16;15:470. doi: 10.1186/s12885-015-1449-9 (PMC4468980; doi:10.1186/s12885-015-1449-9)
Supplement: Additional file 2: Table S2. — Clinical-pathological patients’ characteristics according to WHO grade classification (TCGA). [file 12885_2015_1449_MOESM2_ESM.docx]

**Additional file 2: Table S2** – Clinical-pathological patients’ characteristics according to WHO grade classification

|  | | **Lower Grade Gliomas (LGG)** | | **Glioblastoma Multiforme (GBM)** |
| --- | --- | --- | --- | --- |
| **Variable** | **Category** | **Grade II**  **(N=183)** | **Grade III**  **(N=195)** | **Grade IV**  **(N=567)** |
| Age (years) | Mean±SD | 40.98±13.31 | 45.59±13.17 | 57.92±14.34 |
|  | Median (IQR) | 39 (30-50) | 44 (34-56) | 59 (50-68) |
|  | Range | 14-87 | 22-75 | 10-89 |
| Males (n,%) |  | 96 (52.46) | 112 (57.44) | 348 (61.38) |
| Therapy (n,%) | Missing values (N) | 16 | 15 | 22 |
|  | None | 104 (62.28) | 48 (26.67) | 129 (23.67) |
|  | Radiotherapy only | 33 (19.76) | 23 (12.78) | 56 (10.28) |
|  | Radiotherapy + Chemioteraphy | 30 (17.96) | 109 (60.56) | 360 (66.06) |
| IDH1 (n,%) | Mutated alleles | 104 (56.83) | 90 (46.15) | 14 (2.47) |
| NANOG expression | Mean±SD | 1.64±2.89 | 1.94±3.90 | 0.09±0.05 |
|  | Median (IQR) | 0.88 (0.67-1.46) | 0.96 (0.67-1.56) | 0.08 (0.05-0.11) |
|  | Range | 0.50-24.50 | 0.50-36.64 | 0.01-0.55 |
| TRIM8 expression | Mean±SD | 1.65±2.17 | 1.18±1.70 | 1.94±0.73 |
|  | Median (IQR) | 1.05 (0.63-1.79) | 0.65 (0.32-1.23) | 1.79 (1.46-2.24) |
|  | Range | 0.10-18.16 | 0.10-12.70 | 0.38-5.51 |
| TRIM8 CNV (n° copies) | Missing values (N) | 62 | 58 | 299 |
|  | Loss (-2 copies) | 0 (0.00) | 1 (0.73) | 1 (0.37) |
|  | Loss (-1 copy) | 12 (9.92) | 45 (32.85) | 246 (91.79) |
|  | Normal (2 copies) | 109 (90.08) | 90 (65.69) | 20 (7.46) |
|  | Gain (>2 copies) | 0 (0.00) | 1 (0.73) | 1 (0.37) |
| miR-17 expression | Mean±SD | 414.93±176.47 | 452.24±231.69 | 9.07±0.69 |
|  | Median (IQR) | 393.36 (290.67-494.59) | 401.06 (302.62-552.06) | 9.04 (8.65-9.48) |
|  | Range | 130.44-996.67 | 115.94-2223.02 | 6.04-11.35 |
| Time to progression (months) | Mean±SD | 27.05±34.55 | 21.56±28.54 | 12.19±16.17 |
|  | Median (IQR) | 13.34 (4.20-32.56) | 10.75 (5.25-26.95) | 7.02 (3.25-14.36) |
|  | Range | 0.00-181.84 | 0.00-210.59 | 0.00-127.25 |
| Overall follow-up time (months) | Mean±SD | 27.24±34.59 | 21.79±28.61 | 16.31±17.92 |
|  | Median (IQR) | 13.82 (4.20-32.56) | 10.85 (5.31-26.95) | 11.74 (5.41-19.80) |
|  | Range | 0.00-181.84 | 0.00-210.59 | 0.07-127.25 |
