# Supplementary figures and images for: TRIM8 downregulation in glioma affects cell proliferation and it is associated with patients survival
Source: BMC Cancer. 2015 Jun 16;15:470. doi: 10.1186/s12885-015-1449-9 (PMC4468980; doi:10.1186/s12885-015-1449-9)

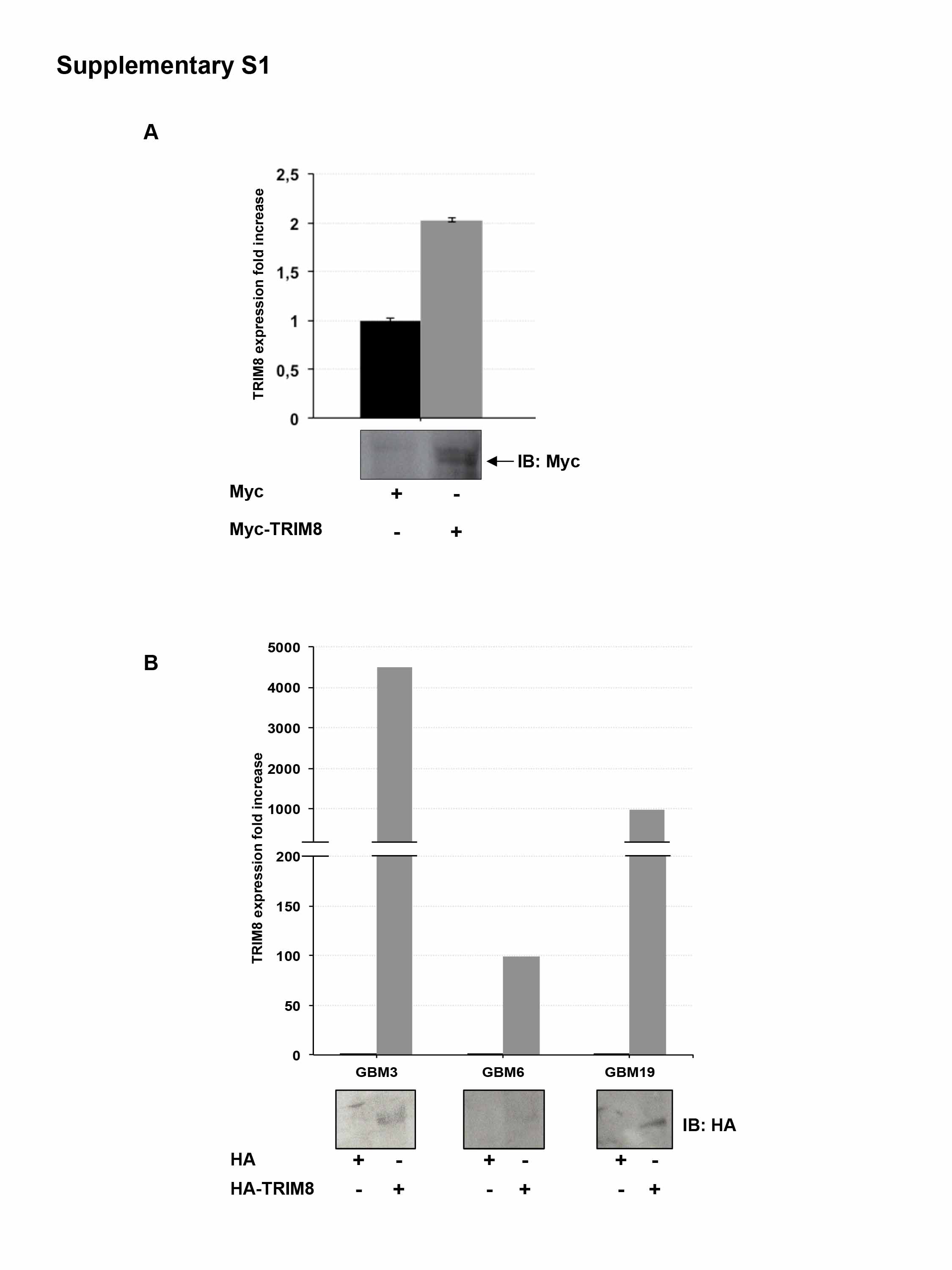

Supplement: Additional file 3: Figure S3. — TRIM8 expression level in transfected U87MG and GBM cells. mRNA and protein level of TRIM8 in U87MG (A) and GBM (B) cell lines transfected with a vector expressing TRIM8 or an empty vector was detected by qPCR (A-B) and Western Blot (A-B), respectively, 72 h post transfection. [file 12885_2015_1449_MOESM3_ESM.jpeg]
